# Do Anxiety Disorders Play a Role in Road Traffic Deaths? The Nested Case-Control Study of Drivers Killed in Fatal Motor Vehicle Accidents in Finland Between 1990 and 2011

**DOI:** 10.1177/00207640261457642

**Published:** 2026-07-10

**Authors:** Tiia Hannila, Pirkko Riipinen, Helinä Hakko, Niina Sihvola, Anu-Helmi Halt

**Affiliations:** 1 Research Unit of Clinical Medicine, University of Oulu, Poulu, Finland; 2 Oulu University Hospital and University of Oulu, Finland; 3 Department of Psychiatry, Oulu University Hospital, Finland; 4 The Finnish Crash Data Institute (OTI), Helsinki, Finland

**Keywords:** anxiety disorder, psychiatric comorbidity, fatal motor vehicle accident, driving-affecting drugs, suicidality

## Abstract

**Background:**

Relatively little is known about the relationship between anxiety disorders and fitness-to-drive. This study analysis the characteristics of motor vehicle fatalities, involving drivers with hospital-diagnosed anxiety disorders, as well as the contribution of psychiatric comorbidity to the cause of these fatalities.

**Aims:**

In our population-based data of all deceased Finnish drivers we compare socio-demographic, driving-related and clinical characteristics of study subjects with anxiety disorders with matched control subjects without any history of hospital treated psychiatric disorders. We also explore whether the cause of death of drivers killed in motor vehicle accidents differs between those with anxiety disorders and comorbid psychiatric disorders.

**Methods:**

This study was based on OTI (The Finnish Crash Data Institute) data with linkage to two national registers: The Care Register for Health Care and the National Cause of Death Register. The initial study population included 4,930 drivers involved in fatal motor vehicle accidents in Finland between 1990 and 2011. It was confirmed that 93 drivers had received an anxiety disorder diagnosis (ICD-10 codes: F40-44) in the 5 years prior to their collision.

**Results:**

We found that drivers with anxiety disorders were more likely to be involved in crashes with suicidal intent (*OR* = 2.83, 95% CI [1.40, 5.72]), drivers who had previously experienced headaches or insomnia (*OR* = 4.99, 95% CI [1.34, 18.62]), and drivers who were impaired by alcohol or drugs (*OR* = 3.92, 95% CI [1.28, 11.98] when DUI was 0.5‰–1.19‰ and *OR* = 2.01, 95% CI [1.15, 3.52] when DUI was aggravated, BAC 1.2‰ or above). It was notable that the proportion of suicides increased when anxiety disorder occurred simultaneously with depression (20.7%) or other psychiatric disorders (24.0%). In the overall dataset, the most common cause of death was an accident (82.8%). Regarding to accidental deaths, no significant differences were observed between individuals with anxiety disorders and matched control subjects without any history of hospital treated psychiatric disorders.

**Conclusion:**

The findings of this study suggest that assessment of suicidal intent may be particularly important when evaluating fitness to drive in patients with anxiety disorders, including those with psychiatric comorbidity, alongside consideration of substance use that may impair driving, and a history of headaches or insomnia.

## Introduction

While research on the impact of psychiatric diseases on driving performance has been conducted, there is still a dearth of evidence-based information and understanding, particularly with regards to the impact of anxiety disorders on drivers of motor vehicles. Driving-related anxiety, as a contributor to risky driving and motor vehicle crashes, has been investigated previously (Zinzow & Jeffirs, 2018). Clapp et al. investigated anxious driving behavior among psychology students using a variety of surveys. They found that anxious driving behavior involves three typical domains: exaggerated safety/caution behaviors, anxiety-based performance deficits and hostile/aggressive driving behaviors (Clapp et al., 2011). Further studies by Clapp et al. found that drivers with anxiety symptoms were identified as being more prone to engage in problematic behavior that increased the risk of car accidents (Clapp et al., 2014). However, previous studies have only investigated anxiety as a symptom in motor vehicle drivers and do not include patient data or statistics on fatal motor vehicle crashes (FMVAs).

Driving-related anxiety can present in several different ways, such as emotional reactivity, threat assessment, physiologic hyperarousal and problematic driving behavior (Zinzow & Jeffirs, 2018). Driving-associated anxiety has also been identified to include disorganized behavior and increased frequency of driving errors and has been linked to many other psychiatric disorders such as specific phobia, post-traumatic stress disorder (PTSD), panic disorder, agoraphobia, social phobia and generalized anxiety disorder. (Clapp et al., 2014). Anxiety disorders often coexist with depression, personality disorders and alcohol or other substance-use disorders (Craske & Stein, 2016).

Research based on nationwide Danish register data established an association between anxiety disorders and excess mortality. The risk of unnatural death (i.e. suicide, accident, homicide), is known to be increased in patients with generalized anxiety disorder, acute stress reaction, PTSD, social phobia or panic disorder. Anxiety disorders not only increase the risk of unnatural death, but also the risk of natural death, that is, the risk of dying from diseases and medical conditions. Unhealthy habits such as physical inactivity increased smoking, poor eating habits, reluctance to seek medical attention or adhere to healthy lifestyle advice along with biological factors can contribute to the increased mortality seen in anxiety disorders. Substance misuse is also more common in patients with anxiety disorders, as anxious individuals may easily turn to drugs or alcohol as a form of self-medication (Meier et al., 2016).

According to World Health Organization, traffic accidents are the leading cause of death, especially in young adults (WHO, 2023). The Finnish Transport and Communications Agency (Finnish Transport and Communications Agency [Traficom], 2021) states that driving health requirements are not met if the person has a serious psychiatric disorder. This includes, for example, acute psychotic disorders and severe antisocial personality disorder combined with substance addiction (Finnish Transport and Communications Agency [Traficom], 2021). Guidelines for doctors on the evaluation of a driver’s abilities, however, appear to be lacking, particularly when it comes to anxiety disorders, about which comparatively little is still known about the potential impact on driving.

In our population-based data of all deceased Finnish drivers we compare socio-demographic, driving-related and clinical characteristics of study subjects with anxiety disorders with matched control subjects without any history of hospital treated psychiatric disorders. We also explore whether the cause of death of drivers killed in motor vehicle accidents differs between those with anxiety disorders and comorbid psychiatric disorders. We had access to the OTI (The Finnish Crash Data Institute) data with linkage to two other comprehensive national registers: The Care Register for Health Care (CRHC) and the National Cause of Death Register. FMVAs analyzed in this study took place in Finland between the years 1990 to 2011 and the information on psychiatric morbidity covered the 10-year-period prior to death in the FMVA.

## Methods

### Data Sources

This study is based on OTI data, with linkage to two other national registers: CRHC and the National Cause of Death Register. We had access to data including all FMVA drivers in Finland between the years 1990 in 2011. The register-linkage was made using personal identity codes, unique to each Finnish citizen (The Finnish Crash Data Institute [OTI], 2022).

The driving and accident data used in this study was collected from the database of the OTI. All FMVAs in Finland are investigated by Finnish Road Accident Teams (RAITs), which consist of experts from various disciplines, including the police, medical professionals, road maintenance and vehicle technology professionals. The main goal of the teams is to improve traffic safety by carefully investigating the factors and risks behind each FMVA, with information collected used to support recommendations for actions required to prevent future accidents. OTI collects the reports prepared by the Investigation teams and records the material in the accident information register (The Finnish Crash Data Institute [OTI], 2022).

The data concerning FMVA drivers’ hospital treatment was provided by CRHC. CRHC provides information on inpatient treatments since 1969 and specialized outpatient visits since 1998. The International Classification of Diseases (ICD) has been used to determine diagnoses. Between the years 1969 and 2011 three different versions of ICD have been in use in Finland: ICD-8 between the years 1969 and 1986, ICD-9 from 1987 to 1995 and ICD-10 since 1996. (Finnish Institute for Health and Welfare, 2022)

The official causes of death and precise dates of deaths were obtained from the Cause of Death Register, maintained by Statistics Finland. In Finland, all suicides and unintentional injuries leading to death are examined by forensic medicine specialist physicians. Official death certificates are completed after the examination and eventually archived by Statistics Finland (Statistics Finland [STAT], 2022).

### Sample Selection

**Figure 1.**
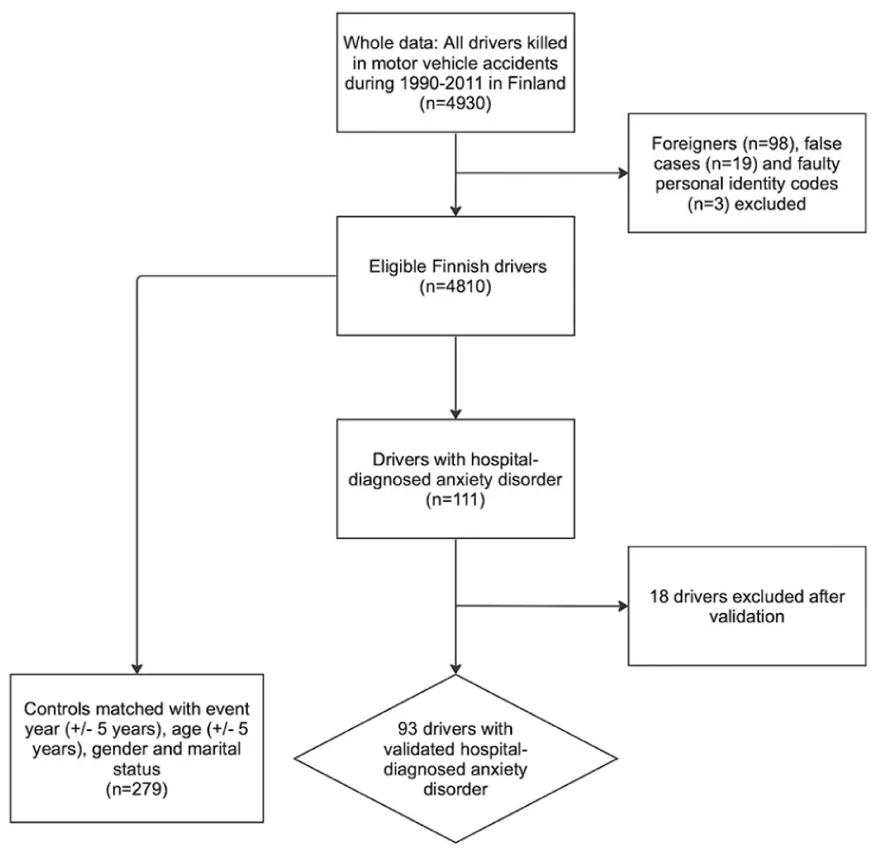
Selection of the study subjects.

Our study focuses on Finnish FMVA drivers with an anxiety disorder diagnosed in hospital during the 5-year period preceding their FMVA. Diagnoses of depression and substance-use disorder (SUD), made during the 10 years before deaths in motor vehicle accidents, were also assessed. Our selection of study subjects is shown in Figure 1.

Two researchers (T.H. & A-H.H.) validated 93 FMVA drivers in Finland between 1990 and 2011 who had been diagnosed with anxiety disorder (ICD-10 codes: F40-44) during the 5 years preceding their fatal accident. Diagnoses of anxiety disorder were based on ICD-8, ICD-9 and ICD-10 classifications. ICD8/9-based diagnoses for anxiety disorder were converted to ICD-10 codes according to the WHO classification. All participants whose anxiety diagnoses was based on single outpatient visits and then later validated to another psychiatric disorder were excluded from the study (*n* = 18).

Drivers were divided into three mutually exclusive subgroups: (1) anxiety disorder without comorbid psychiatric disorder (*n* = 39), (2) anxiety disorder with comorbid depression (*n* = 29), (3) anxiety disorder with other comorbid psychiatric disorder (*n* = 25) e.g. personality disorder (*n* = 13), SUD (*n* = 9), personality disorder and SUD (*n* = 1), unspecified non-organic psychotic disorder (*n* = 1), unspecified psychiatric disorder (*n* = 1).

Of the cases, 78.5% (*n* = 73) were male and 21.5% (*n* = 20) were female. 45.2% (*n* = 42) were unmarried, 23.7% (*n* = 22) were married or cohabiting, 14.0% (*n* = 13) were widowed or divorced, 17.2% (*n* = 16) were other/unknown. The mean (*SD*) age of the cases was 35.1 (13.8) years, the youngest driver was 15.7 years old and the oldest 69.3 years old.

Controls were obtained from the same databases mentioned above. We matched controls with event year (within 5 years of the fatal accident), age (within 5 years of the fatal accident), gender (exact), marital status (unmarried, married, divorced/widow, other/unknown.)

### Study Variables

#### Socio-Demographic Characteristics

Information about each driver’s level of education and employment status was obtained from RAITs (The Finnish Crash Data Institute [OTI], 2022).

Drivers’ level of education and employment status were evaluated as socio-demographic characteristics. Education, either completed or ongoing, was categorized as: comprehensive school, upper secondary school and institution of higher education (university/university of applied sciences education). Employment status consisted of categories for employed, student, unemployed, pensioner and other/unknown.

#### Driving and Accident-Related Characteristics

Data of the driver’s role in the accident, season when the accident occurred, the day of the week of the accident, the main purpose for travel, fellow passengers and driving license were all obtained from the RAITs (The Finnish Crash Data Institute [OTI], 2022).

Drivers were categorized into three groups, depending on the information on the drivers’ role in their FMVAs: at-fault driver in collision, other participant in collision and single vehicle accident. Single vehicle accidents consist of events that do not involve other vehicles, for example, driving off the road.

The season when the accident occurred was derived from the date for each accident. Month of accident was categorized as winter (from November to January), spring (from February to April), summer (from May to June) and autumn (from August to October). The day of the week of the accident was categorized as weekday (Monday–Friday) and weekend (Saturday–Sunday).

The main purpose of travel included categories for work/study related driving, running errands, leisure drive to a planned destination, leisure drive without a planned destination and other/unknown. The type of passengers was categorized as follow: no passengers, family member(s), other persons and unknown.

Driving license was categorized as: yes, suspended driving/driving license expired and no previous driving license/other.

#### Clinical Characteristics

Information on the clinical characteristics was collected from RAITs (The Finnish Crash Data Institute [OTI], 2022).

Use of alcohol and medications affecting driving performance, previous history of insomnia, headache or suicidal behavior were each evaluated as clinical characteristics for each driver.

Instances of driving under the influence of alcohol (DUI) were derived from the variable “blood alcohol concentration (BAC, ‰)”, being measured either from blood or breath samples. Breath samples can be taken if the driver did not die immediately following the accident. BAC was categorized as: no alcohol or under 0.5 ‰/unknown, DUI (BAC, 0.5‰–1.19‰) and aggravated DUI (BAC, 1.2 ‰ or above.) Use of medications affecting driving performance was categorized as yes or no.

Driver’s previous history of insomnia or headache was categorized as no/unknown or yes. Driver’s previous suicidal behavior was categorized as no/unknown or yes.

### Statistical Analyses

Statistical significance of group difference in categorical variables was assessed with the Pearson’s chi-square or Fisher’s Exact test, and in continuous variables, with the Student’s t-test or Mann-Whitney U-test. Logistic regression analysis was used to analyze the association of socio-demographic, driving related, and clinical characteristics of deceased drivers to anxiety disorder in comparison to age-, sex-, accident year and marital status matched control drivers died in the FMVA. The statistical software used in analyses was the IBM SPSS Statistics, version 29.

## Results

### Characteristics of the Study Participants

#### Anxiety Diagnoses of the Study Participants

Anxiety diagnoses of the study participants involved in FMVAs are presented in Table 1. Adjustment disorder (23.7%) and unspecified anxiety disorder (21.5%) were the two largest groups in this study. The third largest group was mixed anxiety disorder and depressive disorder (11.8%).

**Table 1.** Anxiety Diagnoses of Drivers Involved in FMVAs With Anxiety Disorders.

Diagnostic code (ICD-10)	Disorder	*n* (%)
F43.2	Adjustment disorder	22 (23.7)
F41.9	Anxiety disorder, unspecified	20 (21.5)
F41.2	Mixed anxiety and depressive disorder	11 (11.8)
F41.0	Panic disorder	9 (9.7)
F41.1	Generalized anxiety disorder	6 (6.5)
F40.1	Social phobias	6 (6.5)
F40.2	Specific (isolated) phobias	4 (4.3)
F41.3	Other mixed anxiety disorders	3 (3.2)
F43.0	Acute stress reaction	3 (3.2)
F42	Predominantly obsessional thoughts or ruminations	2 (2.2)
F43.1	Post-traumatic stress disorder	2 (2.2)
F40.0	Agoraphobia	1 (1.1)
F43.8	Other reactions to severe stress	1 (1.1)
F43.9	Reaction to severe stress, unspecified	1 (1.1)
F44.2	Dissociative stupor	1 (1.1)
F44.7	Mixed dissociative disorders	1 (1.1)
	Total	93 (100)

#### Socio-Demographic Characteristics

Socio-demographic characteristics are presented in Table 2. There was a statistically significant difference between cases and controls in the level of education (*p* = .049). There were more drivers in the case group who had attended a comprehensive school (64.5%) and an institution of higher education (12.9%) than in controls (comprehensive school 58.3%, institution of higher education 8.2%). There was also a statistically significant difference in the employment status (*p* = .021), with more cases of unemployed (8.6%) or pensioners (12.9%) than in the control group.

**Table 2.** Distribution of Characteristics of Drivers Involved in FMVAs With and Without Anxiety Disorders.

Characteristic	Total	Drivers with anxiety disorders (cases)	Drivers without any psychiatric disorder (controls)	Statistical significance, overall *p*-value
*n* (%)	372	93 (25.0)	279 (75.0)	
Socio-demographic characteristics				
Level of education				.**049**
Comprehensive school	217	60 (64.5)	157 (58.3)	
Upper secondary school	120	21 (22.6)	99 (35.5)	
Institution of higher education	35	12 (12.9)	23 (8.2)	
Employment status				.**021**
Employed	205	48 (51.6)	157 (56.3)	
Student	54	14 (15.1)	40 (14.3)	
Unemployed	20	8 (8.6)	12 (4.3)	
Pensioner	26	12 (12.9)	14 (5.0)	
Other/not known	67	11 (11.8)	56 (20.1)	
Driving-related characteristics				
Driver’s role				.**119**
At-fault driver in collision	172	48 (51.6)	124 (44.4)	
Other participant in collision	51	7 (7.5)	44 (15.8)	
Single vehicle accident	149	38 (40.9)	111 (39.8)	
Season				.**706**
Winter	71	14 (15.1)	57 (20.4)	
Spring	67	17 (18.3)	50 (17.9)	
Summer	120	31 (33.3)	89 (31.9)	
Autumn	114	31 (33.3)	83 (29.7)	
Day of the week				.**853**
Weekday	233	59 (63.4)	174 (62.4)	
Weekend	139	34 (36.6)	105 (37.6)	
Main purpose for travel				.**158**
Work/study related driving	60	11 (11.8)	49 (17.6)	
Running errands	46	7 (7.5)	39 (14.0)	
Leisure drive, planned destination	118	30 (32.3)	88 (31.5)	
Leisure drive, not planned destination	52	14 (15.1)	38 (13.6)	
Other/not known	96	31 (33.3)	65 (23.3)	
Fellow passengers				**1.000**
No passengers	146	52 (80.0)	94 (79.0)	
Family member(s)	12	4 (6.2)	8 (6.7)	
Other person(s)	22	8 (12.3)	14 (11.8)	
Unknown	4	1 (1.5)	3 (2.5)	
Driving license				.**014**
Yes	321	77 (82.8)	244 (87.5)	
Suspended driving/driving license expired	25	12 (12.9)	13 (4.7)	
No previous license/other	26	4 (4.3)	22 (7.9)	
Clinical characteristics				
DUI at time of FMVA				.**003**
No alcohol or under 0.5‰/unknown	243 (65.3)	48 (51.2)	195 (69.9)	
DUI (BAC, 0.5‰–1.19‰)	17 (4.6)	8 (8.6)	9 (3.2)	
Aggravated DUI (BAC, 1.2‰ or above)	112 (30.1)	37 (39.8)	75 (26.9)	
Use of medications affecting driving performance (yes)	33 (8.9)	20 (21.5)	13 (4.7)	.**001**
Previous insomnia or headache (yes)	13 (3.5)	8 (8.6)	5 (1.8)	.**005**
Previous suicidal behavior (yes)	16 (4.3)	14 (15.1)	2 (0.72)	.**001**

Bold formatting is used for table formatting only and does not denote statistical significance.

#### Driving-Related Characteristics

Driving-related characteristics are presented in Table 2. There was a statistically significant difference between cases and controls regarding driving license status (*p* = .014), with 12.9% of cases having a suspended or expired driver’s license, while the corresponding number in the controls was 4.7%. Generally, drivers with anxiety disorder were characterized as being the at-fault driver in collisions (51.6%), the crash occurred in summer or autumn (33.3% on both seasons), usually on a weekday (63.4%), the reason for travel being for leisure with a planned destination (32.2%) or other/not known (33.3%), they drove without passengers (80.0%) and had a valid driving license (82.8%).

#### Clinical Characteristics

Clinical characteristics are presented in Table 2. There was a statistically significant difference in cases exceeding the drink driving limit (BAC, ⩾0.5‰) compared to controls (*p* = .003). Of note was that 48.4% of the drivers with anxiety disorder had illegal levels of blood alcohol (BAC, ⩾0.5‰), while in the control group the corresponding percentage was 30.1%.

According to the RAITs reports, the proportion of drivers who were deemed to have taken medications with the potential to affect driving performance was statistically significantly higher in drivers with anxiety disorder than in controls (*p* = .001). 21.5% of the drivers with anxiety disorder were evaluated to have taken medications that could affect their driving ability, while the corresponding percentage was 4.7% among controls.

Recent insomnia and headache were also found to associate with being a driver with anxiety disorder involved in accidents (*p* = .005). 8.6% of the drivers with anxiety disorder had previously experienced insomnia or headaches, compared to controls, of which 1.8% had previous history of insomnia or headaches.

There was also a statistically significant difference in previous suicidal behavior (*p* = .001). 15.1% of the drivers with anxiety disorder had had previous suicidal behavior, while in the control group 0.7% had a history of suicidal behavior.

### Characteristics Associated With Being a Driver of FMVAs With Anxiety Disorders

Table 3 summarizes the results of logistic regression analysis and shows the statistically significant socio-demographic, driving-related and clinical characteristics associated with a FMVA driver with anxiety disorder, in comparison to controls without any psychiatric disorder.

**Table 3.** Associations of Socio-Demographic, Driving-Related and Clinical Characteristics with the Risk of Involvement in FMVAs in Drivers With Anxiety Disorders, Using Logistic Regression Model.

Characteristic	*OR* [95% CI]	Statistical significance, overall *p*-value
Level of education		.**010**
Comprehensive school	0.65 [0.28, 1.49]	.**305**
Upper secondary school	0.28 [0.11, 0.72]	.**008**
Institution of higher education	REF	
Suicidal intent (yes)	2.83 [1.40, 5.72]	.**004**
Previous insomnia or headache (yes)	4.99 [1.34, 18.62]	.**017**
DUI at time of FMVA		.**008**
No alcohol or under 0.5‰/unknown	REF	
DUI (BAC, 0.5‰–1.19‰)	3.92 [1.28, 11.98]	.**017**
Aggravated DUI (BAC 1.2‰ or above)	2.01 [1.15, 3.52]	.**015**
Use of medications affecting driving performance (yes)	5.10 [2.18, 11.95]	<.**001**

Values shown in bold are statistically significant (p < .05).

A crash with suicidal intent appears to associate with drivers with anxiety disorder involved in fatal crashes (*OR* = 2.83, 95% CI [1.40, 5.72], *p* = .004). Previous history of insomnia or headaches was also associated with drivers with anxiety disorder involved in fatal crashes, with the likelihood of drivers in FMVA having anxiety disorder being almost five-fold (*OR* = 4.99, 95% CI [1.34, 18.62], *p* = .017). Driving under the influence of alcohol associates with drivers involved in FMVA having an anxiety disorder. The likelihood of drivers in FMVA having anxiety disorder was almost four-fold when DUI was 0.5‰–1.19‰ (*OR* = 3.92, 95% CI [1.28, 11.98], *p* = .017) and two-fold when DUI was aggravated (BAC 1.2‰ or above; *OR* = 2.01, 95% CI [1.15, 3.52], *p* = .015). Post-mortems found medications at the time of death also associated with drivers involved in FMVA having anxiety disorder. There was an over five-fold likelihood of drivers with anxiety disorder being involved in a fatal crash when medications with the ability to affect driving performance has been used (*OR* = 5.10, 95% CI [2.18, 11.95], *p* = <.001). In the case of socio-demographic characteristics, having an upper secondary school level of educational reduced the risk of drivers with anxiety disorder being involved in FMVAs (*OR* = 0.28, 95% CI [0.11, 0.72], *p* = .008).

Only those factors that remained statistically significant in the logistic model are reported in the table.

### Anxiety Disorders and Psychiatric Comorbidity

The differences between the psychiatric comorbidity groups are presented in Table 4. The official causes of death were obtained from the Cause of Death Register. There was a statistically significant difference in death category between study groups (*p* = .003). In all subgroups, the most common cause of death was an accident. It is also noticeable that the proportion of suicides is higher in the comorbid depression (20.7%) and other psychiatric disorder’s group (24.0%) than anxiety disorders without comorbid psychiatric disorders (5.1%). There was also a statistically significant difference in the drivers’ role (*p* = .049). There were statistically significantly more at-fault drivers in the group of anxiety disorders with comorbid depression (72.4% of the drivers) than in any other group.

**Table 4.** Official Cause-of-Death Category and Driver’s Role in FMVAs in Drivers with Anxiety Disorder With and Without Psychiatric Comorbidity and Controls.

Characteristic	Total	Anxiety disorder without comorbid psychiatric disorder	Anxiety disorder with comorbid depression	Anxiety disorder with other comorbid psychiatric disorder	Drivers without any psychiatric disorder (controls)	Statistical significance, overall *p*-value
*n* (%)	372	39 (10.5)	29 (7.8)	25 (6.7)	279 (75.0)	
Death category						.**003**
Disease	12 (3.23)	0 (0.0)	1 (3.5)	0 (0.0)	11 (3.9)	
Accident	308 (82.80)	35 (89.8)	18 (62.0)	17 (68.0)	238 (85.3)	
Suicide	30 (8.06)	2 (5.1)	6 (20.7)	6 (24.0)	16 (5.7)	
Undetermined intent	22 (5.91)	2 (5.1)	4 (13.8)	2 (8.0)	14 (5.0)	
Driver’s role						.**049**
At-fault driver in collision	172 (46.24)	16 (41.0)	21 (72.4)	11 (44.0)	124 (44.4)	
Other participant in collision	51 (13.71)	5 (12.8)	1 (3.5)	1 (4.0)	44 (15.8)	
Single vehicle accident	149 (40.05)	18 (46.2)	7 (24.1)	13 (52.0)	111 (39.8)	

Values shown in bold are statistically significant (p < .05).

## Discussion

We investigated drivers with a hospital-diagnosed anxiety disorder who were killed in motor vehicle accidents in Finland between the years 1990 and 2011. We defined socio-demographic, clinical and driving-related characteristics of FMVA drivers with an anxiety disorder. We also compared death category and the driver’s role in relation to comorbid diagnoses with depression or other psychiatric disorders, such as personality disorders and SUD. The major findings of our study are that drivers with anxiety disorders were more likely to be involved in a FMVA if the crash was driven with suicidal intent, the driver had previously suffered from insomnia or headaches, or the driver was driving under the influence of alcohol or drugs. Anxiety disorder is also a significant factor in traffic suicides especially if anxiety disorder occurs simultaneously with depression or other psychiatric disorders.

We found that crashes with suicidal intent associated with FMVAs involving drivers with a hospital-diagnosed anxiety disorder. Suicidal intent as the reason for collision has markedly increased in drivers involved in FMVA between 2002 and 2011 (Sassi et al., 2018). It was also noteworthy that 27% of drivers with anxiety disorder involved in FMVA were classified as “undetermined” when assessing driver’s intent. In drivers with comorbid depression, a significant proportion of drivers were classified as at-fault driver in collisions (72%), while a high percentage (14%) of deaths death were categorized as undetermined. It is possible that suicide rates amongst drivers are underestimated. Indeed, studies have shown that is often difficult to determine whether a FMVA was due to suicide or unintentional intent (Hernetkoski & Keskinen, 1998; Wyatt et al., 2009). It is possible therefore, that our study underestimates the levels of suicidal intent in drivers with anxiety disorder involved in FMVAs.

We also found that use of alcohol and/or medication with the potential to affect driving, at the time of death, were both risk factors that associated with being a driver with anxiety disorder involved in a FMVA. For example, benzodiazepines are widely prescribed for the treatment of anxiety disorders and insomnia, despite being associated with impaired driving skills (Bandelow et al., 2017). It is known that substance-misuse is also more common in patients with anxiety disorders, as anxious individuals may easily turn to drugs or alcohol as a form of self-medication (Meier et al., 2016). These findings must be considered whenever evaluating a patient with anxiety disorder’s ability and fitness to drive.

Anxiety disorders are known to be associated with sleep disturbances and poor sleep (Cox & Olatunji, 2016). Drivers who suffer from insomnia are more prone distraction and high risk driving (Zhang et al., 2023). In our study, FMVA drivers with anxiety disorder had a fivefold likelihood of suffering from insomnia and/or headaches compared to controls without an anxiety disorder. Sleep disorders and tiredness at the wheel have been identified as risk factors for traffic accidents (Kalsi et al., 2018). For this reason, when evaluating a patient with anxiety disorder’s capacity to drive, it’s also important to consider their physical health, particularly with regard to a history of headaches and insomnia.

In our study, the majority of drivers with anxiety disorder involved in FMVAs were classified as the at-fault driver in collisions. With the exception of those without any comorbid psychiatric disorder, the most common accident classification was single vehicle accident. The most common cause of death in all subgroups was accident. Drivers with anxiety symptoms have been identified as being more prone to engage in problematic behavior that increases the risk of traffic accidents (Clapp et al., 2014). Anxiety disorders have also been identified to associate with attention problems such as selective attention to negative information. (Cabrera et al., 2020). Driving in traffic requires a high level of concentration, and for this reason, we are considering whether biased attention to information has an effect on driving ability in patients with anxiety disorders.

Individuals suffering from anxiety disorders are anxious, fearful or try to avoid perceived threats in the environment. Anxiety disorders are characterized by a disproportionate reaction to actual risk or danger. To be diagnosed with an anxiety disorder, fear and anxiety must be significant and persistent and be associated with impairment in functioning (Craske & Stein, 2016). In our study, there were two large diagnostic subgroups in FMVA drivers with an anxiety disorder. Almost half of the drivers with an anxiety disorder had an adjustment disorder (23.7%) or unspecified anxiety disorder (21.5%). Unspecified anxiety disorder diagnoses often lead to underdiagnosis, inaccuracies and undertreatment. Patients are frequently assumed to have an unspecified anxiety disorder diagnosis even if they meet the criteria for a specific anxiety disorder on a structured diagnostic interview. This can result for example in poorer health outcomes (Fletcher et al., 2019). The diagnosis of adjustment disorders can also be challenging, as the current DSM- and ICD-criteria are vague and the measures are sometimes incomplete. Furthermore, adjustment disorders form a clinically very heterogeneous group, with varying symptomatology, such as anxiety, depression, and behavioral changes (Zelviene & Kazlauskas, 2018).

Depression, personality disorders, and SUDs frequently coexist with anxiety disorders (Craske & Stein, 2016). Suicidality rose considerably in our study when an anxiety disorder coexisted with co-morbid depression or another psychiatric disorder, such as SUD or personality disorder. 21% of drivers with comorbid depression and 24% of drivers with other comorbid psychiatric disorders died by suicide, while the corresponding number for drivers with anxiety disorder without any comorbid psychiatric disorder was 5%.

While road accidents are the leading cause of death, particularly for young adults (World Health Organization [WHO], 2023) anxiety disorders are the most prevalent group of psychiatric illnesses and typically manifest in early adulthood (Penninx et al., 2021). In Finland, guidelines for a physician’s evaluation of a driver’s ability have been established by Traficom. Traficom deems that driving health requirements are not met if the person has a serious psychiatric disorder such as acute psychotic disorder. There are insufficient guidelines for doctors regarding the evaluation of capacity to drive in patients with anxiety disorders. Remarkably, 83% of the drivers with anxiety disorders possessed a current driver’s license at the time of their vehicle accident; although a significant number of the cases (15%) had a history of suicidal behavior. It is, therefore, critical to thoroughly consider suicidal intention and risk in individuals with anxiety disorders when determining their fitness to drive, particularly when their anxiety coexists with depression or other comorbid psychiatric disorders.

### Strengths and Limitations

A major strength of our study was the use of two comprehensive national registers, which allowed examination of all FMVA drivers in Finland during a 10-year study period. The linkage between these two registers was possible using unique personal identification codes given to all Finnish residents. Combined data from OTI database, the Care register for Health Care and the Cause of Death Register enabled overall analysis of characteristics of the FMVA drivers with and without an anxiety disorder. The accident data, provided by the Finnish Crash Data Institute, is internationally unique in relation to the amount and quality of the data. Their road traffic accident data is used in both national and international research (The Finnish Crash Data Institute [OTI], 2022).

Our study’s primary weakness stems from the relatively small number of cases included. This limited our ability to classify anxiety disorders precisely and, thus, prevented us from making recommendations about health guidelines for various different anxiety disorders. Accordingly, the findings should be interpreted cautiously, and any conclusions should be considered tentative. A further limitation is that we were unable to assess the precise pharmacological profile of the drivers at the time of the FMVA because, except for alcohol, we were not in possession of pharmacological data for drug or substance use. In addition, we were not able to reliably assess the risk of non-suicidal collisions due to limitations in the data. Since physicians in Finland are only required to report driving restrictions to the police for periods longer than 6 months, our data did not include information on shorter driving bans. Unfortunately, there was not statistical power enough to compare persons to other anxiety disorder groups or analyze the severity of anxiety disorder.

## Conclusion

The findings of this study suggest that anxiety disorders may be important to consider when assessing fitness to drive among psychiatric patients, and that the assessment of suicidal intent may be particularly important in patients with anxiety disorders. More than half (57%) of drivers with anxiety disorders involved in fatal crashes had received one of the following diagnoses: adjustment disorder, unspecified anxiety disorder or mixed anxiety and depressive disorder. This suggests that accurate and early diagnosis of anxiety disorders may be important in clinical work. The increase in adjustment disorders in the data is also interesting, as it appears to be associated with more severe outcomes, such as fatal crashes. When evaluating a patient with adjustment disorder, it would be important to consider the assessment of psychosocial support more broadly in addition to the assessment of driving ability. When prescribing medications with the potential to affect a person’s ability to drive, we recommend that doctors consider the patient’s fitness-to-drive and clearly warn against driving while being under the influence of the drug. Careful evaluation of fitness to drive should be considered in patients with anxiety disorders, particularly in the presence of psychiatric comorbidity, suicidal ideation or behavior, substance use that may impair driving, or a history of headaches or insomnia.
